# Shedding Light on the Microbial Community of the Macropod Foregut Using 454-Amplicon Pyrosequencing

**DOI:** 10.1371/journal.pone.0061463

**Published:** 2013-04-23

**Authors:** Lisa-Maree Gulino, Diane Ouwerkerk, Alicia Y. H. Kang, Anita J. Maguire, Marco Kienzle, Athol V. Klieve

**Affiliations:** 1 Rumen Ecology Unit, Department of Agriculture, Forestry and Fisheries, Queensland, Dutton Park, Queensland, Australia; 2 School of Agriculture and Food Sciences, University of Queensland, Gatton, Queensland, Australia; 3 Centre for Animal Science, Queensland Alliance for Agriculture and Food Innovation, St. Lucia, Queensland, Australia; Dowling College, United States of America

## Abstract

Twenty macropods from five locations in Queensland, Australia, grazing on a variety of native pastures were surveyed and the bacterial community of the foregut was examined using 454-amplicon pyrosequencing. Specifically, the V3/V4 region of 16S rRNA gene was examined. A total of 5040 OTUs were identified in the data set (post filtering). Thirty-two OTUs were identified as ‘shared’ OTUS (i.e. present in all samples) belonging to either Firmicutes or Bacteroidetes (Clostridiales/Bacteroidales). These phyla predominated the general microbial community in all macropods. Genera represented within the shared OTUs included: unclassified Ruminococcaceae, unclassified Lachnospiraceae, unclassified Clostridiales, *Peptococcus* sp. *Coprococcus* spp., *Streptococcus* spp., *Blautia* sp., *Ruminoccocus* sp., *Eubacterium* sp., *Dorea* sp., *Oscillospira* sp. and *Butyrivibrio* sp. The composition of the bacterial community of the foregut samples of each the host species (*Macropus rufus*, *Macropus giganteus* and *Macropus robustus*) was significantly different allowing differentiation between the host species based on alpha and beta diversity measures. Specifically, eleven dominant OTUs that separated the three host species were identified and classified as: unclassified Ruminococcaceae, unclassified Bacteroidales, *Prevotella* spp. and a *Syntrophococcus sucromutans*. Putative reductive acetogens and fibrolytic bacteria were also identified in samples. Future work will investigate the presence and role of fibrolytics and acetogens in these ecosystems. Ideally, the isolation and characterization of these organisms will be used for enhanced feed efficiency in cattle, methane mitigation and potentially for other industries such as the biofuel industry.

## Introduction

Macropods are marsupials belonging to the family Macropodidae that includes kangaroos and wallabies. Macropods are herbivorous and can be divided into grazers (feed on perennial native grasses/sedges), browsers (feed on small shrubs) and mixed feeders (both grazers and browsers). The stomachs of grazing macropods have an enlarged forestomach region, the sacciform and tubiform, where microbial fermentation of ingested plant material takes place and the hind-stomach, which secretes hydrochloric acid and pepsinogen [1]. In common with other foregut fermenting herbivores, the sacciform and tubiform regions possess a consortium of microbes that are involved in enteric fermentation [2], [3]. Although there are many similarities between macropod foregut microbial fermentation and fermentation in the rumen of ruminants, e.g. sheep and cattle, it has been demonstrated that there are significant differences in the digesta flow and digestion/absorption patterns [1]. It has been proposed that differences in digestion may occur due to physiological differences in the gut structure and the process of selection may have an effect on the microbial population [1].

Very few studies have examined the bacterial community of the macropod foregut. Dellow et al. [3] enumerated foregut bacteria in wild *Macropus giganteus* (Eastern grey kangaroo) and *Thylogale thetis* (Red-necked pademelon) and captive *T. thetis* and *Macropus eugenii* (Tammar wallaby) macropods but did not attempt to characterise the organisms present. Most studies involving taxonomic classifications of bacteria present in the macropod foregut have focused on those that can be cultured in the laboratory [4] and/or those involved in reductive acetogenesis [5], [6]. Macropods produce significantly less methane (or no methane) than cattle [7], [8] and this appears to be due, at least in part, to the dominance of reductive acetogenesis in these animals [5]. Therefore the identification and isolation of reductive acetogens has been explored in terms of a potential methane mitigation strategy to reduce emissions from domestic ruminants (sheep and cattle).

Use of conventional molecular technologies, such as clone libraries and microarrays, to attempt to unravel complex microbial ecosystems, like those found in the foregut of herbivores, is time consuming, costly and can lack sensitivity only identifying dominant bacterial populations. Developments in high throughput pyrosequencing, specifically targeted gene sequencing, provides a means of overcoming these limitations enabling the in depth analysis of complex microbial ecosystems [9]. Several studies have utilized 454-amplicon pyrosequencing to examine the microbial communities present in herbivorous foregut fermenters such as cattle, sheep, and hoatzin [10], [11], [12]. Very little molecular work however, has been performed on the macropod foregut microbial communities and the importance that this microbiome may have in mitigating greenhouse gas emissions and improving the efficiency of fermentation of poor quality grasses. Pope et al. [13] has examined the foregut bacterial community (specifically using the 16S rRNA gene and cloning/Sanger sequencing) of Tammar wallabies (*Macropus eugenii*). A single paper by Muegge et al. [14] has used 454-amplicon pyrosequencing to examine the faecal microbial communities in a range of mammals, which included a captive *M. rufus* sample which grouped with other herbivorous foregut fermenters (gazelle, springbok, giraffe, sheep and okapi - [14]). With so little being known and understood of the bacterial populations that normally inhabit the foregut of macropods, this study used 454-amplicon pyrosequencing to examine the bacterial communities in the foregut of 20 wild large grazing macropods from Queensland, Australia. In addition to examining the bacterial diversity that exists in macropod forestomach contents the variability within these ecosystems in relation to geographical location, diet and host species were also examined.

## Materials and Methods

### Samples and Sample Preparation

Samples consisted of archival DNA extracted from forestomach content samples obtained from twenty wild macropods including *Macropus giganteus, Macropus rufus* and *Macropus robustus*, collected from five locations across Queensland in 2007. The sampling locations were chosen based on the dominant native pasture present within that region; the location, dominant pasture species, and number and species of kangaroo collected from are contained in Table 1. Samples were collected under the Queensland Parks and Wildlife Scientific Purposes Permit number WO/001397/00/SSA. As macropod samples were from a ‘wild’ population and were collected as part of a national cull, animal ethics approval was not required, however, work was conducted and complied as per conditions governed in the permit (WO/001397/00/SSA). Original sample collection details, storage and DNA extraction protocols were as described previously [5], [15]. Briefly, foregut fluid samples were collected (approximately 5–15 g) and a 1 ml aliquot of foregut fluid was placed into a clean eppendorf tube and stored on ice until transfer to the laboratory. Aliquots were then centrifuged at 13,200 rpm for 10 minutes and the pellet retained and stored at −20°C until ready for DNA extraction. DNA was extracted from the pelleted sample; all samples were extracted within one week of each other, and DNA was stored at −20°C. DNA concentrations were calculated using the Nanodrop 8000 Spectrophotometer (Thermoscientific, Australia) as per manufacturer’s instructions. Samples were diluted with dH_2_O to a final concentration of 10 ng/µL and used as template in subsequent barcoded PCR.

**Table 1:** Macropod forestomach samples: host, pasture and location details.

Sample name	Abbreviation	Species	Sex	Predominant pasturecommon name (species name)	Abbreviation	Location	Abbreviation
**Wallaroo Buck 9**	WB9	*Macropus robustus*	Male	Mitchell grass *(Astrebla* spp.)	MG	Charleville	Ch
**Grey Doe 10**	GD10	*Macropus giganteus*	Female	Mitchell grass *(Astrebla* spp.)	MG	Charleville	Ch
**Grey Buck 11**	GB11	*Macropus giganteus*	Male	Mitchell grass *(Astrebla* spp.)	MG	Charleville	Ch
**Red Doe 12**	RD12	*Macropus rufus*	Female	Mitchell grass *(Astrebla* spp.)	MG	Charleville	Ch
**Wallaroo Doe 14**	WD14	*Macropus robustus*	Female	Mitchell grass *(Astrebla* spp.)	MG	Charleville	Ch
**Wallaroo Doe 15**	WD15	*Macropus robustus*	Female	Mitchell grass *(Astrebla* spp.)	MG	Longreach	Lr
**Grey Doe 17**	GD17	*Macropus giganteus*	Female	Mitchell grass *(Astrebla* spp.)	MG	Longreach	Lr
**Red Doe 19**	RD19	*Macropus rufus*	Female	Mitchell grass *(Astrebla* spp.)	MG	Longreach	Lr
**Red Buck 20**	RB20	*Macropus rufus*	Male	Mitchell grass *(Astrebla* spp.)	MG	Longreach	Lr
**Wallaroo Buck 21**	WB21	*Macropus robustus*	Male	Spinifex (*Spinifex* spp.)	Spin	Cloncurry	Cl
**Red Buck 22**	RB22	*Macropus rufus*	Male	Spinifex (*Spinifex* spp.)	Spin	Cloncurry	Cl
**Wallaroo Doe 23**	WD23	*Macropus robustus*	Female	Spinifex (*Spinifex* spp.)	Spin	Cloncurry	Cl
**Red Buck 24**	RB24	*Macropus rufus*	Male	Spinifex (*Spinifex* spp.)	Spin	Cloncurry	Cl
**Red Doe 25**	RD25	*Macropus rufus*	Female	Spinifex (*Spinifex* spp.)	Spin	Cloncurry	Cl
**Grey Doe 30**	GD30	*Macropus giganteus*	Female	Black Speargrass *(Heteropogon contortus)*	Bck	Charters Towers	CT
**Grey Buck 31**	GB31	*Macropus giganteus*	Male	Bluegrass *(Dicanthium sericeum)*	BlG	Dingo	Di
**Grey Doe 32**	GD32	*Macropus giganteus*	Female	Bluegrass *(Dicanthium sericeum)*	BlG	Dingo	Di
**Grey Buck 35**	GB35	*Macropus giganteus*	Male	Bluegrass *(Dicanthium sericeum)*	BlG	Dingo	Di
**Wallaroo Doe 37**	WD37	*Macropus robustus*	Female	Bluegrass *(Dicanthium sericeum)*	BlG	Dingo	Di
**Wallaroo Buck 38**	WB38	*Macropus robustus*	Male	Bluegrass *(Dicanthium sericeum)*	BlG	Dingo	Di

### Barcoded PCR and 454-amplicon Pyrosequencing

Samples for 454-amplicon pyrosequencing were amplified in triplicate, using a barcoded universal 16S rRNA gene (variable regions V3/V4) forward 341F (5′ Adapter A – Barcode - TACGGGAGGCAGCAG - 3′ (Life Sciences, 2009, [16]) and reverse 787R (5′ Adapter B - CTACCAGGGTATCTAAT - 3′ modified from [17] primer pairs. PCR mixtures were prepared to a final volume of 50 µL with the following reagents and final concentrations: 1 Unit of Phusion polymerase (Finnzymes, Australia); 250 mM of (each) forward and reverse primers; 200 nM dNTP mix (Roche, Australia); 1 X’s HF Phusion Buffer and 20 ng of template DNA. Thermocycling was performed in an eppendorf mastercycler (Eppendorf, Australia) using the following conditions: - lid preheated to 101°C; initial denaturation 98°C for 30 sec; followed by 30 cycles of: 98°C, 10 sec; 65°C, 20 sec; 72°C, 15 sec; followed by a final extension step at 72°C, 10 min and a ‘cooling’ step at 12°C, 5 min. Products were visualised on a 2% agarose gel post stained with 3 Xs gel red (Jomar Scientific, Australia). Amplicons of correct size (approximately 520 bp) were excised using GelX 6.5 excision tips (Cleaver Scientific, UK) and purified using the QIAquick gel extraction kit (QIAGEN, Australia) as per manufacturer’s instructions, with a modification to the final elution step, eluting the product in 30 µL instead of the recommended 50 µL. Purified PCR product was quantified using the Qubit fluorometer (Invitrogen, Australia) as per manufacturer’s instructions. Approximately 300 ng of purified PCR product for each of the 20 samples was sent to the Australian Genome Research Facility (AGRF) for 454-amplicon pyrosequencing.

### Pyrotag Handling and Analysis

Accurate quantification of the barcoded amplicons using BioAnalyzer, pooling and library preparation (including emulsion PCR using Titanium chemistry) and 454-amplicon pyrosequencing was performed by AGRF. Data was processed using Roche Newbler Software (version 2.5.3, Roche) and returned as raw data in a standard flowgram file (.sff) format. Raw data (.sff) was converted to.fasta and.qual files using mothur [18] and denoised using Acacia software [19]. Denoised sequences and metadata were submitted to MG-RAST (http:/metagenomics.anl.gov) and are stored under project name ‘QEMH’ - record numbers for each amplicon library are: 4513090.3 (WD15), 4513089.3 (WD14), 4513083.3 (RD12), 4513085.3 (RD25), 4513082.3 (RB24), 451309.1 (WD23), 4513081.3 (RB22), 4513073.3 (GB11), 4513086.3 (WB21), 4513080.3 (RB20), 4513084.3 (RD19), 4513077.3 (GD17), 4513076.3 (GD10), 4513087.3 (WB38), 4513075.3 (GB35), 4513079.3 (GD32), 4513088.3 (WB9), 4513074.3 (GB31) and 4513078.3 (GD30). Denoised sequences were analysed using the Quantitative Insights Into Microbial Ecology (QIIME) pipeline software (version 1.5.0) [20]. Specifically, sequences were deconvoluted into individual samples based on their barcodes and simultaneously filtered to remove poor/low quality sequences. Sequences with a quality value of less than 25 were discarded, across a sliding window of 50bp, and sequences between 430bp –470bp in length were retained. Sequences from samples were clustered using Uclust into Operational Taxomonic Units (OTUs) based on 97% sequence similarity and the most abundant sequence within an OTU was chosen as the OTU’s representative sequence. The representative sequences were then aligned against the 16S rRNA Greengenes core set ([21] - http://greengenes.lbl.gov/) using PyNAST [20]. Representative sequences were taxonomically classified using the Ribosomal Database Project (RDP) classifier [22] from the Phylum to the Genus level, using Greengenes Taxonomy. An OTU table was constructed; chimeras identified using Chimera Slayer [23] and removed from the OTU table (as were low confidence singletons - single sequences that occurred in less than two samples). A representative OTU phylogenetic tree and taxonomic summary from Phylum to Genus level were constructed using the QIIME built-in scripts including the fasttree method for tree construction. Alpha diversity indices (Shannon-Weiner, Phylogenetic Distance, equitability and number of observed species) were calculated at a sequence depth of 8,700 sequences as the nearest number (in multiples of 100) to the minimum number of sequences for a sample (8,747). Beta diversity measures (PCoA) were also calculated using unweighted and weighted UniFRAC distances at a depth of 8700 sequences.

### Data Analysis Using R/RStudio

The data was analysed in RStudio: integrated development environment for R Version 0.96.122 (http://www.rstudio.org). Heatmaps were generated using the package “gplots” and the heatmap.2 function of OTU abundance tables (e.g. raw number of sequences/total number of sequences for a sample, x 100) of shared OTUs, i.e. OTUs that were present in all samples. A transformation-based Principal Components Analysis (tb-PCA) [24] using the Hellinger distance was used to ordinate the 20 samples according to OTU abundance data. This dataset contains more than 5,000 OTUs (>5,000 OTUs), which is difficult to represent graphically in a meaningful way. Therefore only the OTUs for which variation of abundance contributed most to the clustering obtained from the tb-PCA were selected and represented in the tb-PCA. The OTUs were identified using the largest contributors, representing the top 0.01% of OTUs, to the first two principal components as selection criteria.

## Results

### Sampling Depth, Coverage and Alpha Diversity

**Figure 1:**
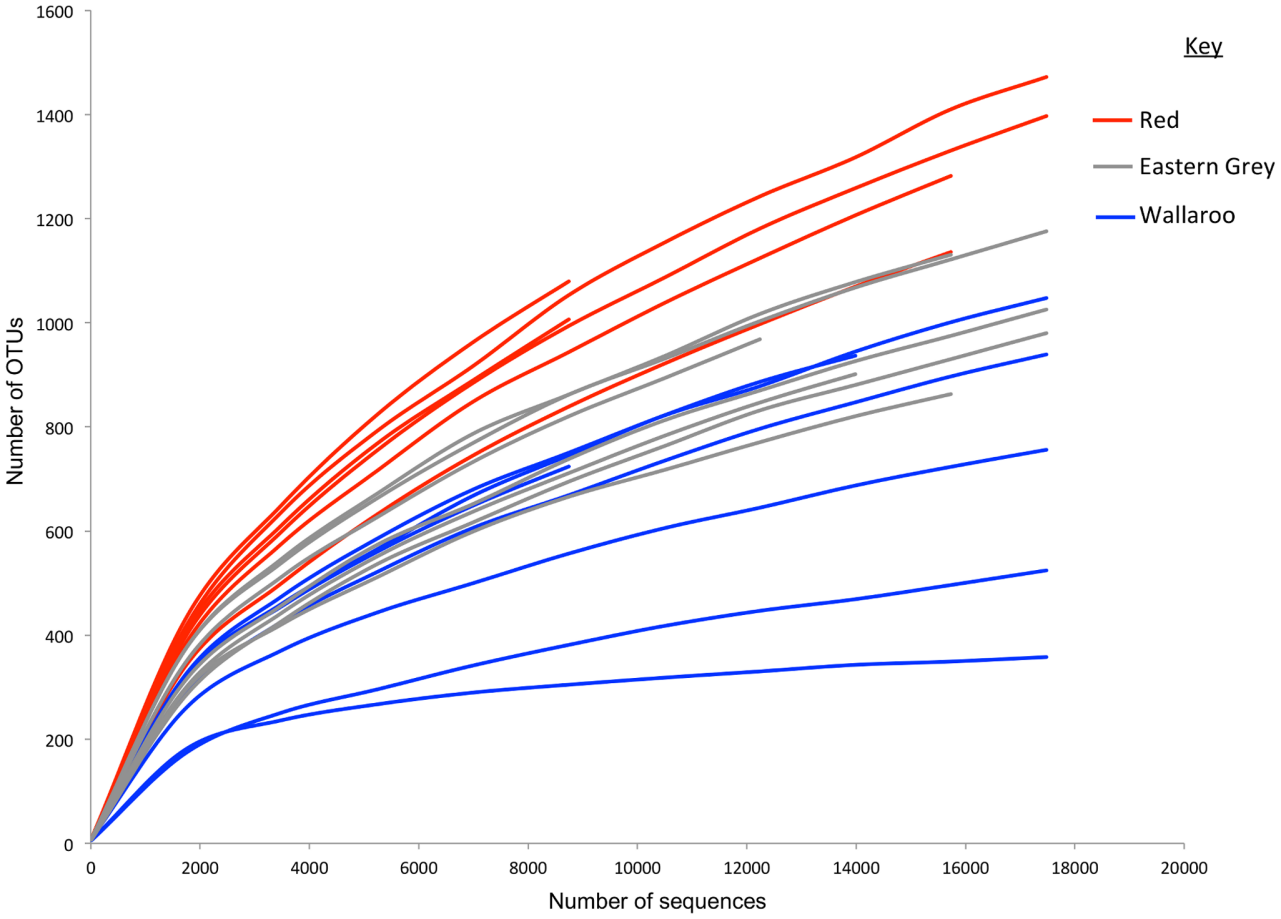
Rarefaction analysis of partial bacterial 16S rRNA gene sequences in macropod foregut samples. Rarefaction was conducted at a genetic distance of 3%. The Red – Red Kangaroo (*Macropus rufus*); Eastern Grey – Eastern Grey Kangaroo (*Macropus giganteus*); Wallaroo – *Macropus robustus* forestomach samples are marked by the red, grey and blue colours respectively.

A total of 350,438 sequences remained post-filtering including chimera and low confidence singleton removal and 5,040 OTUs were identified at the 97% similarity level. Sequence number per sample ranged from 8,747 sequences (WD23) to 26,171 sequences (WD14) with a mean of 17,521±4,666 (mean ± SD; *N* = 20) sequences per sample (Table S1). Rarefaction was performed at the OTU level, and rarefaction curves demonstrated firstly: diversity differed between individual samples (no. OTUs observed), and secondly: the majority of bacterial diversity (OTUs) was not uncovered, as many of the plots were not close to becoming asymptotic (Figure 1). Alpha diversity measures (Phylogenetic Distance (PD); Shannon-Wiener index (Shannon); equitability/evenness; Simpson index; number of OTUs observed) are presented for each host species in Table 2.

**Table 2:** Measures of alpha diversity of bacterial communities for each species of macropod forestomach samples from Queensland calculated at a depth of 8700 sequences.

Species	Phylogenetic diversity	Number of OTUs/Observed species	Shannon	Equitability	Simpson
***Macropus robustu*** **s**	45.66^a^	595^a^	6.04^a^	0.66	0.93
***Macropus giganteus***	53.04^b^	760^b^	6.68^a, b^	0.70	0.96
***Macropus rufus***	62.3^c^	992^c^	7.29^b^	0.74	0.97

Means with different subscript letter (per column) are significantly different from each other (p≤0.05) by protected LSD.

Alpha diversity measures indicated that *M. rufus* forestomach samples had significantly higher bacterial diversity than the other host species according to: PD and number of OTUs (Table 2). Conversely, the forestomach samples from *M. robustus* were the least diverse of the host species according to the same measures (Table 2). The equitability index and Simpson index did not differ significantly between the host species.

### Taxonomy of the Bacteria Present in the Macropod Foregut

**Figure 2:**
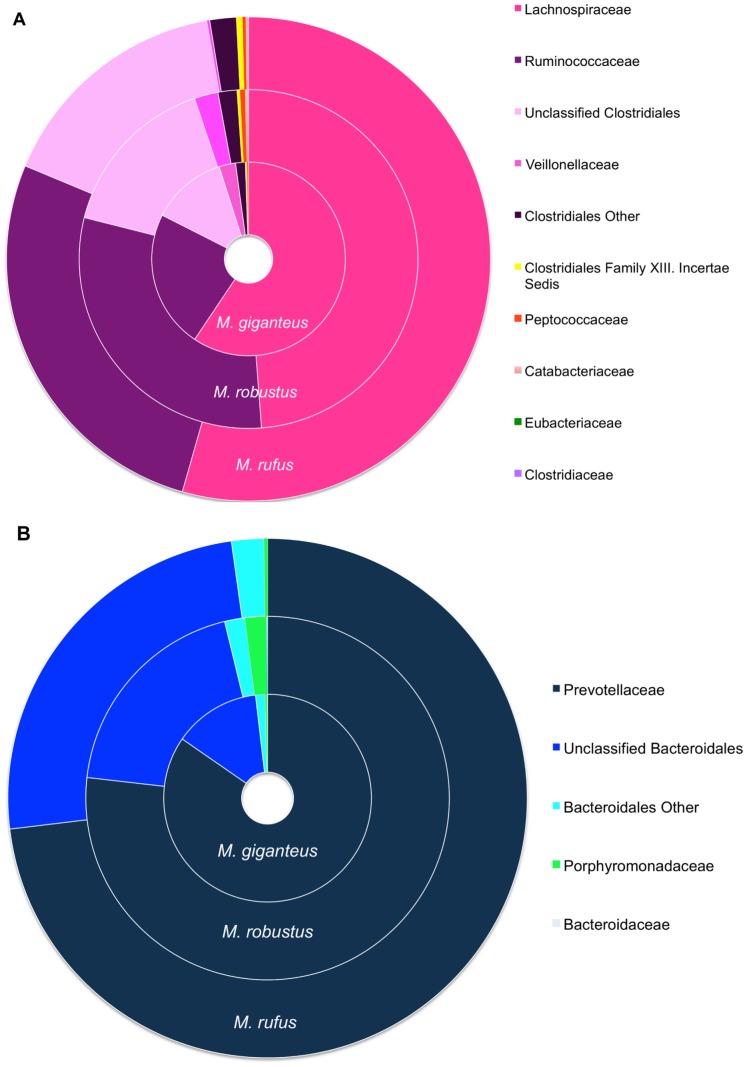
Percent abundance of members of: A) Clostridiales B) Bacteroidales in the foregut of macropods. The percentage abundance of A) Clostridiales B) Bacteroidales identified in the foregut of 20 Queensland macropods using 454-amplicon pyrosequencing.

Ten phyla were identified and, listing them in descending order of mean abundance for all samples, were: – Bacteroidetes (48.3% ±9.19), Firmicutes (47.3% ±9.85), Proteobacteria (1.0% ±1.21), Fibrobacteres (1.0% ±1.19), Fusobacteria (0.9% ±1.28), Actinobacteria (0.6% ±0.367), Spirochaetes (0.4% ±0.334), and (Other –0.4% ±0.229– composed of OTUs that could not be classified at the Phylum level), Tenericutes (0.1% ±0.111) and SR1 (<0.1% ±0.002). Table 3 displays the average Phyla levels for each macropod species. At deeper taxonomic levels, 20 classes, 36 families, 57 genera and 5,040 OTUs were identified. The phyla Fibrobacteres, Fusobacteria, ‘Other’ phylum and SR1 were each composed of a single class and order. Bacteroidetes, Firmicutes, Proteobacteria, Actinobacteria, Spirochaetes, and Tenericutes were composed of two or more classes. Bacteroidia and Clostridia were the two most predominant classes (48.3% ±9.19, 46.2% ±10.32 abundance respectively). Bacteroidia and Clostridia were composed of two orders Bacteroidales (48.3% ±9.19), Flavobacteriales (0.005% ±0.008); and Clostridiales (46.1% ±10.3), Clostridia other (0.005% ±0.006) respectively. As Bacteroidales and Clostridiales comprised over 90% of the total bacterial foregut community present in macropod foregut samples, families within these two predominant orders are presented in Figure 2 (A - Clostridiales, B - Bacteroidales). Prevotellaceae (37.95% ±11.44) and Lachnospiraceae (25.06% ±7.30) were the dominant families respectively.

**Table 3:** Phyla abundance of bacterial OTUs present in each macropod species.

	% of sequences					
Phylum	*M. giganteus*	±SD	*M. robustus*	±SD	*M. rufus*	±SD
**Bacteroidetes**	48.05	0.090	53.76	0.085	42.26	0.072
**Firmicutes**	46.05	0.097	42.64	0.101	54.26	0.065
**Fibrobacteres**	1.53	0.016	0.27	0.003	1.11	0.010
**Fusobacteria**	1.45	0.014	0.67	0.016	0.44	0.004
**Proteobacteria**	1.37	0.013	1.29	0.014	0.20	0.001
**Actinobacteria**	0.66	0.006	0.54	0.003	0.73	0.001
**Spirochaetes**	0.45	0.002	0.36	0.004	0.36	0.004
**Unclassified Bacteria**	0.41	0.001	0.42	0.002	0.48	0.003
**Tenericutes**	0.02	0.000	0.06	0.001	0.16	0.001
**SR1**	≤0.00	0.000	≤0.00	0.000	≤0.00	0.000

**Figure 3:**
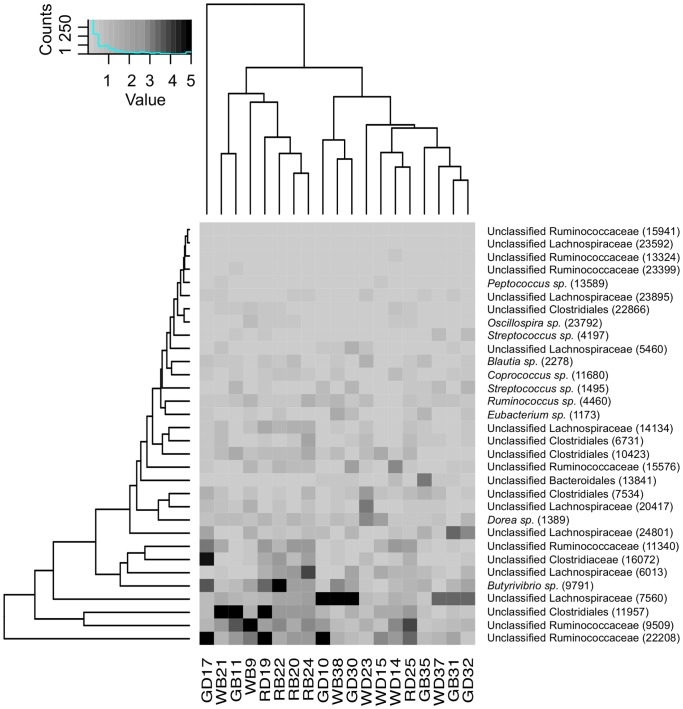
OTU Heatmap with double hierarchical clustering of the shared bacterial OTUs of macropod foregut. OTU Heatmap with double hierarchical clustering of the shared bacterial OTUs of macropod foregut, as determined by 454-amplicon pyrosequencing. Samples are listed along the horizontal axis of the heatmap (GD – Grey Doe; GB – Grey Buck; WD – Wallaroo Doe; WB – Wallaroo Buck; RD – Red Doe; RB – Red Buck), and OTU taxonomy and OTU number (in parentheses) along the vertical axis. Shading is graduated with darker shading indicative of OTUs present in high abundance. Of the 32 shared OTUs identified, one OTU (13841) was classified as a Bacteroidetes, the remainder were classified as Firmicutes.

At 97% sequence similarity, 5,040 OTUs were identified. Of the 5,040 OTUs, 32 were present in all samples and were identified as shared OTUs (Figure 3). A single shared OTU was identified as belonging to the phylum Bacteroidetes (unclassified Bacteroidales), whereas the remaining OTUs (n = 31) were classified as Firmicutes. Within the Firmicutes, two OTUs were classified as Bacilli (*Streptococcus* spp.) and the remaining OTUs (n = 29) were classified as Clostridia. Within the Clostridia, OTUs were either identified as unclassified Clostridia, members of Lachnospiraceae or Ruminococcocaeae. OTUs that could be classified at the genus level include: *Blautia*, *Coprococcus*, *Dorea*, *Eubacterium*, *Peptococcus*, *Oscillospira* and *Ruminococcus*. Seven of the OTUs identified had significant sequence identity (>99% - NCBI BLAST nucleotide database) to bacteria and clones from macropod hosts with three OTUs related to *M. eugenii* clones, two OTUs related to *M. rufus* clones and one OTU related to a *M. giganteus* bacterial isolate (Table S2). The remainder of the OTUs had high sequence identity (≥98%) to bacterial isolates and clones from the gastrointestinal tract of various animals including: horse, sheep, pigs, mice, cattle and humans (Table S2).

### Intra-sample Diversity (Beta Diversity) of the Macropod Foregut Bacterial Community

**Figure 4:**
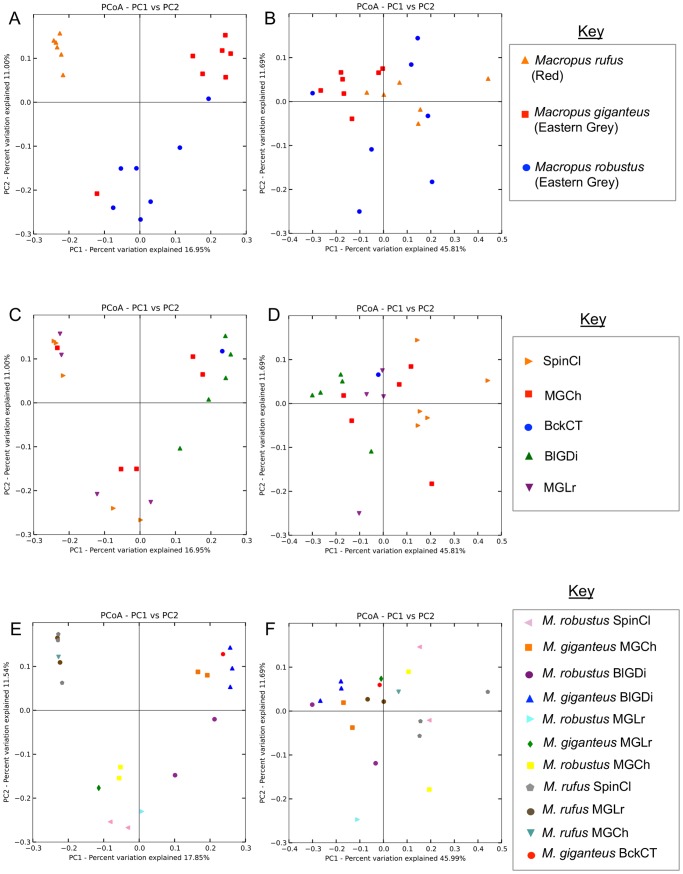
Principal Co-ordinate Analysis (PCoA) of bacterial OTUS from the macropod foregut of 20 macropods. Principal Co-ordinate Analysis (PCoA) of bacterial OTUS from the macropod foregut of 20 macropods from Queensland. A) Unweighted PCoA by host species B) Weighted PCoA by host species C) Unweighted PCoA by feedtype/location D) Weighted PCoA by feedtype/location E) Unweighted PCoA by species/feedtype/location F) Weighted PCoA by species/feedtype/location. SpinCl = Spinifex/Cloncurry, MGCh = Mitchell grass/Charleville, BckCT = Blackgrass/Charters Towers, BlGDi = Bluegrass/Dingo, MGLr = Mitchellgrass/Longreach.

Unweighted and weighted clustering using Principal Coordinates Analysis (PCoA) of Unifrac distance matrices were performed, and labeled according to the following variables: host; location/predominant feedtype (treated as a single variable) and host/location/predominant feedtype (treated as a single variable). Unweighted PCoA demonstrated that the host species is the primary variable in the dataset (Figure 4A), as most samples tended to cluster together based on the host species. However, no clustering was evident based on the weighted PCoA of host species (4B), nor on the unweighted or weighted PCoA of feedtype/location (Figures 4C and 4D). Clustering was evident based on the unweighted PCoA of host/feedtype/location combination (Figure 4E). No clustering was apparent for the weighted PCoA by host/feedtype/location combination (Figure 4F).

**Figure 5:**
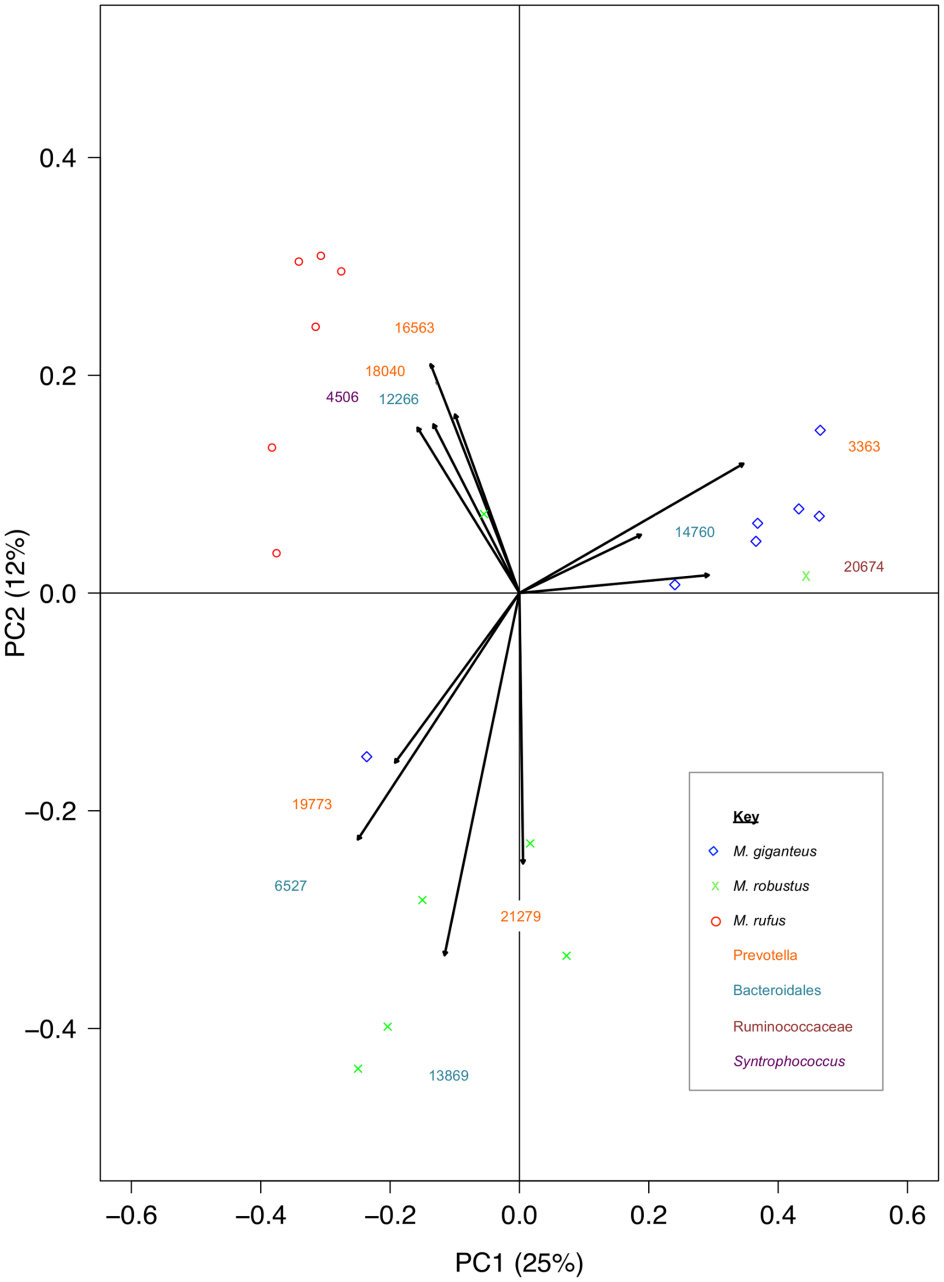
Transformation based Principal Component Analysis (tb-PCA) of bacterial OTUs from 20 Queensland macropod foregut samples. The top 0.01% OTUs contributing to the separation of samples are displayed on the figure (OTU number). OTUs were classified as *Prevotella spp.*, Bacteroidales (unclassified), Ruminococcaceae (unclassified) and *Syntrophococus spp*.

Transformation based Principal Components Analysis (tb-PCA) was used to identify the variable that accounts for the most variation in groups of samples. The top 0.01% of OTUs contributing to the separation of species was identified and plotted (Figure 5). Three main clusters were observed that loosely based on host species with the exception of two *M. robustus* samples that clustered with different host species (*M. giganteus* and *M. rufus*) and a single *M. giganteus* sample that was very loosely associated with the bulk of the *M. robustus* samples. The four OTUs contributing to the separation of *M. rufus* from other species were classified as *Prevotella* spp., Bacteroidales and a *Syntrophococcus* sp. Three OTUs were identified as contributing to the separation of *M. giganteus* and were classified as *Prevotella* sp., Ruminococaceae (unclassified) and Bacteroidales (unclassified). Likewise, for the separation of *M. robustus*, four OTUs were identified (unclassified Bacteroidales and *Prevotella* spp.). Five of the eleven OTUs were highly homologous (≥97%) to clones from *M. eugenii* foregut. A single OTU had 98% identity to the acetogen *Syntrophococcus sucromutans* (S185) isolated from the rumen of cattle. The remaining OTUs (n = 5) had identity of ≤94% to uncultured clones from a variety of herbivorous animals (rhinoceros, elephant) and an omnivore, the pig-like babirusa representing novel families (Table S3).

## Discussion

### Introduction

This study has revealed a diverse and complex bacterial community within the forestomach of large grazing macropods and is the first study, using 454-amplicon pyrosequencing, to comprehensively examine this specific ecosystem.

### Sample Coverage

Rarefaction curves demonstrated that the sequencing depth of this study did not uncover the majority of the bacterial diversity within the kangaroo foregut. As this was the first study specifically using 454-amplicon pyrosequencing to examine macropod foregut bacterial communities, it was difficult to predetermine what was an adequate sequencing depth that enabled sufficient coverage. In analogous herbivorous foregut fermenters (e.g. sheep, cattle, gazelle, springbok) sequencing depths ranging from 1,700–20,000 sequences have been adequate for uncovering diversity [14], [25], [26], [27]. Future work could aim to have a greater depth of sequencing to improve coverage to the level suggested (90% coverage) by Lemos et al. [28] for pyrosequencing of the 16S rRNA gene.

### Gross Diversity at Phyla and Family Level

Results revealed that Firmicutes and Bacteroidetes were the dominant Phyla in all animals examined. This result is consistent with Ley et al. [29] and Pope et al. [13] where it was established that Firmicutes and Bacteroidetes were the predominant Phyla identified in *M. rufus* faeces and the *M. eugenii* foregut. It is not surprising that Firmicutes and Bacteroidetes are the predominant Phyla identified, as they have been found to dominate in many herbivorous gut systems [26], [27], [30]. However, the proportions reported Ley et al. [29] and Pope et al. [13] varied considerably to our findings with Firmicutes representing approximately 60% and Bacteroidetes approximately 20% in both studies. Consequently, the Firmicutes to Bacteroidetes ratio (FB ratio) that occurred in our study was close to 1∶1 (varied 0.78–1.26), whilst in the aforementioned studies it was closer to 3∶1. The gut FB ratio has been linked in humans to several variables including: age, obesity and diet [31], [32], [33]. Mariat et al. [32] discovered a low FB ratio was associated with infants (3 weeks to 10 months old) and older people (70−90 years old) as opposed to a high FB ratio, which was found adults (20–45 years old). Whereas Ley et al. [31] found that high FB ratio was associated with obese people and De Fillipo et al [30] found that a higher FB ratio occurred in European children (diet consists of high animal protein and low fibre content) compared to children from Burkina Faso (low FB ratio, on a high fibre, low fat diet). It is possible that any of these variables could explain the differences in FB ratios reported in our samples, as could the host species, diet or sample type (faeces vs. gut sample). Therefore, future work could be performed to examine multiple variables and their effect on the gut FB ratios in macropods.

At the deeper levels of Class and Order, Clostridia/Clostridiales and Bacteroidia/Bacteroidales contributed to over 85% of the total bacterial community. This result is consistent with other herbivorous fermenters including ruminants [26], [34], [35], [36], [37], Tammar wallaby [13] and Galapagos Island iguanas [29] where Bacteroidia/Clostridia were found in high abundance in all samples examined. The high abundance of Bacteroidia/Clostridia may be a common feature of herbivorous fermenters and could warrant further investigation. Within the Orders Clostridiales and Bacteroidales, the families Prevotellaceae, Ruminococcaceae and Lachnospiraceae predominated respectively (Figure 2). The same families have been observed to be predominant and common organisms in the cattle rumen (based on 16S rRNA gene data), regardless of diet, breed and age [26], [34], [35], [36], [37]. The presence of these organisms in macropods suggests that they may be performing similar functions as occurs in the rumen (i.e. fibre digestion, degradation of proteins and polysaccharides, production of volatile fatty acids and protein and nutrient recycling [37], [38]). Future work could utilise newer molecular technologies, such as metatranscriptomics, to elucidate the functions and metabolic pathways present in the macropod foregut.

### Shared OTUs and Roles

Shared OTUs from this study were identified as OTUs that were found in all macropods examined. Firmicutes and Bacteroidetes were the only Phyla represented as shared OTUs (Figure 3). Within Firmicutes, many of the OTUs were identified as unclassified Lachnospiraceae, unclassified Ruminococcaceae and unclassified Clostridiales. It is not possible to deduce the exact function of these novel groups in each system based on 16S rRNA data alone however many culture-independent techniques (using 16S rRNA) examining the gut bacterial communities have identified the same ubiquitous groups in macropods *M. eugenii* and *M. rufus* [13], [29]; cattle [25], [26], [27]; mice [39], [40] and human [41], [42], [43] gut ecosystems. Interestingly, only ten shared OTUs could be classified at the genus level and included a *Coprococcus* sp. that displayed high identity (>99%) to an OTU present in a captive *M. rufus* feacal sample [29]. The presence of these homologous sequences in our data, suggests that these particular species may be performing the same function in macropods regardless of the host species, as well as performing similar functions as to what occurs in other gut ecosystems. However, functional assays in tandem with phylogenetic screening would need to be performed to further substantiate this claim.

The function of shared OTUs that could be identified to the species level (based on ≥98% identity, Greengenes named isolates – Table S2) can be speculated based on cultured representatives of these species. Of particular interest are shared OTUs identified in our study that have highly homologous 16S rRNA gene sequences to fibrolytic bacteria found in the rumen environment such as OTU #4460 (*Ruminococcus flavefaciens*) and OTU #9791 (*Butyrivibrio fibrisolvens*). Both *R. flavefaciens* and *B. fibrosolvens* have been demonstrated as members of a consortium of microbes involved in fibrolytic digestion (specifically hemicellulose and xylan deconstruction) in the rumen [44], [45], [46], [47], [48], [49], [50], [51]. It is not surprising that fibrolytic bacteria, similar to those that occur in the rumen, were identified in macropods, particularly as many macropods in Northern Australia are often co-grazing on the same native pastures as beef/dairy cattle [52]. The identification of fibrolytic bacteria is important for many industries worldwide including the beef cattle industry. Improved fibrolytic activity in the rumen can result in improved feed efficiency and animal growth, which would be beneficial to the cattle industry [53]. Specifically the use of exogenous enzymes applied to feed prior and with ingestion, has shown improved growth of cattle [54], [55], [56], [57]. Glycosyl hydrolases (GH) are another useful group of enzymes that are capable of breakdown of cellulosic biomass and are of significant interest to the biofuel industry [58]. Rumen metagenomes have been scoured to identify potential GH genes [59]. It is highly likely that novel and uncharacterized fibrolytic bacteria and hence novel cellulolytic enzymes exist in macropods. Future work will focus on generating metagenomes and metatranscriptomes of our samples for bio-prospecting purposes.

### Alpha Diversity

Differences were observed in alpha diversity measures for individual samples (Table S1). The host species *M. rufus* had the highest diversity of all the species examined and differed significantly from the other host species (Table 2). Studies by Ley et al. [29], Muegge et al. [14] and Pope et al., [13] are the only comparable studies published that have examined the general bacterial community of macropod foreguts using molecular techniques, specifically targeting the 16S rRNA gene. Ley et al. [29] and Muegge et al. [14] examined faecal samples of captive *M. rufus* as part of broader studies of gut ecosystems (Ley et al. [29] (n = 2, Sanger sequencing) and Muegge et al. [14] (n = 1, 454-amplicon pyrosequencing)); and Pope et al. [13] a captive *M. eugenii (*n = 8, Sanger sequencing). The average number of observed species obtained for the aforementioned studies was 111 [29], 870 [14] and 206 [13]. Numbers of OTUs observed in our study were much higher (average 1050), however this may be a reflection of sequencing depth achieved (thousands for pyrosequencing as compared to hundreds for traditional cloning/sequencing), rather than a reflection of the actual diversity. Alpha diversity measures must be interpreted with caution, as it has been demonstrated that sequence number can have an affect on alpha diversity measures (especially observed species). Additionally, alpha diversity measures that utilize rare species (i.e. singletons and doubletons) in their calculations (such as Good’s coverage and Chao) are not robust across varying sampling/sequence depths [28].

### Beta Diversity

Beta diversity (PCoA and tb-PCA) supported the differentiation of the macropod host species based on the gut bacterial community (Figure 4 and Figure 5) and this is the first report, demonstrating clear differences in the macropod bacterial foregut community. Eleven dominant OTUs were identified that contributed to this separation and five OTUs were highly homologous (>97%) to isolates from *M. eugenii* and four of these OTUs were classified as novel *Prevotella* spp. (Figure 5 and Table S2). The reported roles of many species of *Prevotella* specifically in the rumen of cattle and sheep (where they tend to predominate) are varied and broadly cover the degradation of starch, proteins and peptides [60], [61], [62], [63] and although not cellulolytic organisms *per se*, many species are known to produce cellulolytic enzymes [61]. The exact role of novel *Prevotella* spp. in macropods remains to be elucidated.

Another dominant OTU of interest that separated most of the *M. giganteus* samples from the other macropod species displayed high identity to a ruminal *Syntrophococcus sucromutans* that is a known reductive acetogen. Identifying a reductive acetogen in macropods is not surprising, as it has been proposed that hydrogenotrophic bacteria (specifically reductive acetogens) utilise hydrogen and reduce carbon dioxide to acetate via the reductive acetogenesis pathway [4]. However, in the rumen, methane is generated by methanogenic archaea that utilise hydrogen and reduce carbon dioxide and this is thought to out-compete reductive acetogenesis. It is unexpected that a reductive acetogen with high similarity to a ruminal acetogen was identified in a macropod sample. Furthermore, to complicate the reductive acetogenesis/methanogenesis issue, homologous 16S rRNA gene sequences from previously identified macropod reductive acetogens [4], [5], were identified in our study (data not shown). Therefore it is essential that the identification and role of reductive acetogens in macropods be further understood. This could be achieved by direct comparison of macropod and rumen gut samples.

### Future Work/conclusions

As many novel organisms are present in macropods, it is important that future work is geared towards linking the phylogeny of novel organisms to their function. Manefield et al. [64] used RNA Stable Isotope Probing (SIP), in conjunction with Denaturant Gradient Gel Electrophoresis (DGGE) to identify and classify phenol-degrading bacteria in an industrial bioreactor. A similar approach (either DNA or RNA SIP) may be used to identify hydrogenotrophic bacteria in the macropod foregut utilizing deuterium (^2^H) or ‘heavy’ carbon dioxide (^13^CO_2_) as SIP substrates. New technologies such as metagenomics and metatranscriptomics could also be used to establish metabolic functions in parallel with single cell genomics and Fluorescent Activated Cell Sorting (FACS) for identifying and characterizing specific organisms involved in fibre digestion and hydrogenotrophy. Furthermore, the sampling of a wider range of macropods (species and location), combined with the sampling of analogous ecosystems (e.g. ruminants) should enable the elucidation of metabolic pathways, and organisms involved that are shared between the ecosystems and are discordant between the ecosystems. Ideally, the identification of fibrolytics will be exploited for improved feed efficiency and subsequently, animal growth of commercial ruminants and the identification and characterization of hydrogenotrophs (specifically reductive acetogens) utilized in agricultural methane mitigation.

## Supporting Information

Table S1 **Measures of Good’s estimate and alpha diversity of bacterial communities present in 20 wild macropod forestomach samples from Queensland calculated at a depth of 8700 sequences.** GB = Grey Buck, GD = Grey Doe, RB = Red Buck, RD = Red Doe, WB = Wallaroo Buck, WD = Wallaroo Doe.
(DOC)

Table S2 **Shared bacterial OTUs present in all 20 wild macropod forestomach samples from Queensland, Australia, classified using the BLAST algorithm against the Greengenes and NCBI Genbank nucleotide databases.**
(DOC)

Table S3 **Bacterial OTUs, identified using tb-PCA, that enable the separation of 20 wild macropod forestomach samples into host macropod species, classified using the BLAST algorithm against the Greengenes and NCBI Genbank nucleotide databases.**
(DOC)
