# Supplementary material for: Shedding Light on the Microbial Community of the Macropod Foregut Using 454-Amplicon Pyrosequencing
Source: PLoS One. 2013 Apr 23;8(4):e61463. doi: 10.1371/journal.pone.0061463 (PMC3634081; doi:10.1371/journal.pone.0061463)
Supplement: Table S1 — Measures of Good’s estimate and alpha diversity of bacterial communities present in 20 wild macropod forestomach samples from Queensland calculated at a depth of 8700 sequences. GB = Grey Buck, GD = Grey Doe, RB = Red Buck, RD = Red Doe, WB = Wallaroo Buck, WD = Wallaroo Doe. (DOC) [file pone.0061463.s001.doc]

|  | **Based on raw sequences** | | **Calculated at a depth of 8700 sequences** | | | | | | |
| --- | --- | --- | --- | --- | --- | --- | --- | --- | --- |
| **SampleID** | **Number of sequences** | **Number of OTUs** | **Good's estimate** | **PD** | **Number of OTUs** | **Shannon** | **Simpson** | **Singletons** | **Equitability** |
| GB11 | 14632 | 921 | 0.5 | 53.33 | 704 | 5.99 | 0.93 | 349 | 0.63 |
| GB31 | 13745 | 1025 | 0.48 | 59.14 | 832 | 6.89 | 0.97 | 432 | 0.71 |
| GB35 | 15929 | 869 | 0.5 | 47.49 | 662 | 6.49 | 0.96 | 331 | 0.69 |
| GD10 | 21211 | 1120 | 0.5 | 51.74 | 735 | 6.67 | 0.97 | 371 | 0.7 |
| GD17 | 22045 | 1088 | 0.52 | 45.09 | 666 | 6.01 | 0.94 | 317 | 0.64 |
| GD30 | 20269 | 1249 | 0.54 | 58.24 | 865 | 7.46 | 0.99 | 401 | 0.76 |
| GD32 | 17460 | 1189 | 0.52 | 56.25 | 853 | 7.28 | 0.98 | 409 | 0.75 |
| RB20 | 18021 | 1418 | 0.47 | 61.28 | 1014 | 7.42 | 0.98 | 534 | 0.74 |
| RB22 | 10089 | 1167 | 0.47 | 62.32 | 1079 | 7.51 | 0.98 | 576 | 0.75 |
| RB24 | 10091 | 1078 | 0.47 | 64.4 | 1019 | 7.55 | 0.99 | 535 | 0.76 |
| RD12 | 17100 | 1332 | 0.47 | 60.6 | 949 | 7.48 | 0.99 | 499 | 0.76 |
| RD19 | 24182 | 1706 | 0.47 | 64.97 | 1047 | 7.48 | 0.98 | 556 | 0.75 |
| RD25 | 16610 | 1168 | 0.47 | 60.4 | 844 | 6.31 | 0.92 | 444 | 0.65 |
| WB9 | 18721 | 1085 | 0.49 | 54.85 | 745 | 6.48 | 0.95 | 382 | 0.68 |
| WB21 | 15702 | 987 | 0.53 | 51.66 | 760 | 6.7 | 0.97 | 358 | 0.7 |
| WB38 | 19026 | 782 | 0.54 | 46.64 | 568 | 6.2 | 0.96 | 261 | 0.68 |
| WD14 | 26171 | 1127 | 0.51 | 48.74 | 686 | 6.2 | 0.94 | 336 | 0.66 |
| WD15 | 23170 | 597 | 0.53 | 35.1 | 373 | 4.5 | 0.85 | 175 | 0.53 |
| WD23 | 8747 | 724 | 0.51 | 48.8 | 724 | 6.8 | 0.97 | 354 | 0.72 |
| WD37 | 17517 | 358 | 0.75 | 33.83 | 307 | 5.39 | 0.9 | 78 | 0.65 |
