# Supplementary material for: Shedding Light on the Microbial Community of the Macropod Foregut Using 454-Amplicon Pyrosequencing
Source: PLoS One. 2013 Apr 23;8(4):e61463. doi: 10.1371/journal.pone.0061463 (PMC3634081; doi:10.1371/journal.pone.0061463)
Supplement: Table S2 — Shared bacterial OTUs present in all 20 wild macropod forestomach samples from Queensland, Australia, classified using the BLAST algorithm against the Greengenes and NCBI Genbank nucleotide databases. (DOC) [file pone.0061463.s002.doc]

|  |  | **Greengenes taxonomy** | | | | | **NCBI Genbank BLAST results** | | | | |
| --- | --- | --- | --- | --- | --- | --- | --- | --- | --- | --- | --- |
| **OTU ID** | **Taxonomic Classification** | **Accession number** | **Closest named relative** | **Max identity (%)** | **Host** | **Reference** | **Accession number** | **Closest match** | **Max identity (%)** | **Host** | **Reference** |
| 1495 | *Streptococcus sp.* | GU561372.1 | *Streptococcus sanguinis str. TTE17* | 98.20 | *Homo sapiens* (Oral cavity) | Unpublished | JQ465193.1 | Uncultured bacterium clone 070045_246 | 98 | *Homo sapiens*  (skin) | Unpublished |
| 4197 | *Streptococcus sp.* | FM252031.1 | *Streptococcus suis str. SC84* | 97.53 | *Homo sapiens* (meningitis) |  | [CP003736.1](http://www.ncbi.nlm.nih.gov/nucleotide/402808029?report=genbank&log$=nucltop&blast_rank=3&RID=7PP9ZD7S014) | *Streptococcus suis* S735 | 98 | *Sus scrofa*  *(*pneumonia) |  |
| 2278 | *Blautia sp.* | AB571656.1 | *Blautia coccoides str. JCM 1395* | 97.38 | *-* | Unpublished | [JN884243.1](http://www.ncbi.nlm.nih.gov/nucleotide/363546786?report=genbank&log$=nucltop&blast_rank=1&RID=7PUYNC6X016) | Uncultured bacterium clone 134_OIs2-E10 | 98 | *Sus scrofa*  (ileal mucosa) |  |
| 11680 | *Coprococcus sp.* | AB361624.1 | *Coprococcus catus str. L8* | 98.10 | *Homo sapiens (faeces)* | Unpublished | [GQ137537.1](http://www.ncbi.nlm.nih.gov/nucleotide/253767430?report=genbank&log$=nucltop&blast_rank=1&RID=7PV3PMH9016) | Uncultured bacterium clone 01f02 | 99 | *Sus scrofa*  (waste feed for ASBR reactor) | Unpublished |
| 1389 | *Dorea sp.* | NZ_AAXA02000014.1 | *Dorea formicigenerans str. ATCC 27755* | 99.52 | *Homo sapiens (colon)* | Unpublished | [HQ792022.1](http://www.ncbi.nlm.nih.gov/nucleotide/319499161?report=genbank&log$=nucltop&blast_rank=1&RID=7PVZAHH1014) | Uncultured organism clone ELU0125-T311-S-NI_000263 | 99 | *Homo sapiens* (gastrointestinal specimen) |  |
| 1173 | *Eubacterium sp.* | FP929043.1 | *Eubacterium rectale T/104/1* | 99.05 | *-* | Unpublished | [FJ681740.1](http://www.ncbi.nlm.nih.gov/nucleotide/223686037?report=genbank&log$=nucltop&blast_rank=1&RID=7PXMRB9K016) | Uncultured bacterium clone 5-8E18 | 99 | *Bos taurus*  (faeces) |  |
| 9791 | *Butyrivibrio sp.* | AMO39822.1 | *Butyrivibrio fibrisolvens str. Mz3* | 98.10 | *Bos Taurus* |  | [AY442826.1](http://www.ncbi.nlm.nih.gov/nucleotide/38374192?report=genbank&log$=nucltop&blast_rank=1&RID=7PXVUZEY01N) | Bacterium YE62 | 99 | *Macropus giganteus*  (forestomach content) |  |
| 4460 | *Ruminococcus flavefaciens* | *AF030447.1* | *Ruminococcus flavefaciens str. 007* | 99.29 | *Alces sp.*  *Moose rumen* | Unpublished | [EU719250.1](http://www.ncbi.nlm.nih.gov/nucleotide/190148436?report=genbank&log$=nucltop&blast_rank=1&RID=7R73XPXZ01N) | Uncultured rumen bacterium clone 814353 | 99 | *Bos taurus* Holstein  (rumen) | Unpublished |
| 13589 | *Peptococcus sp.* | X55797.1 | *Peptococcus niger* | 93.03 | *-* |  | [EU776085.1](http://www.ncbi.nlm.nih.gov/nucleotide/192984063?report=genbank&log$=nucltop&blast_rank=1&RID=7PY225TX01N) | Uncultured bacterium clone HY1_d09 | 96 | *Crocuta crocuta* (faeces) |  |
| 23792 | *Oscillospira sp.* | *AB238598.1* | *Oscillibacter valericigenes str. Sjm18-20* | 92.89 | *Japanese corbicula clams (alimentary canal material)* |  | [FJ493113.1](http://www.ncbi.nlm.nih.gov/nucleotide/218988896?report=genbank&log$=nucltop&blast_rank=1&RID=7R70TM9M016) | Uncultured bacterium clone C26 | 99 | *Equus ferus caballus*  (faeces) | Unpublished |
| 13841 | Unclassified Bacteroidales | EU281854.1 | *Eubacterium sp. str F1* | 88.54 | *Bos sp.*  (Cattle rumen) | Unpublished | EU776418.1 | Uncultured bacterium clone KO2_aai19g07 | 92 | *Macropus rufus*  (faeces) |  |
| 16072 | Unclassified Clostridiaceae | AY960565.1 | *Clostridium sp. str. ID11* | 96.42 | *Rattus sp.*  *(faeces)?* |  | [EU461415.1](http://www.ncbi.nlm.nih.gov/nucleotide/169276890?report=genbank&log$=nucltop&blast_rank=1&RID=7PVV9BT3016) | Uncultured bacterium clone KO2_aai20g07 | 99 | *Macropus rufus*  (faeces) |  |
| 6731 | Unclassified Clostridiales | FJ848364.1 | *Clostridium sp. str. P4-6* | 88.57 | Peat | Unpublished | [GU103252.1](http://www.ncbi.nlm.nih.gov/nucleotide/310838724?report=genbank&log$=nucltop&blast_rank=1&RID=7PT3JNF4014) | Uncultured bacterium clone HFV01_172 | 99 | *Homo sapiens*  (faeces) |  |
| 7534 | Unclassified Clostridiales | FJ848364.1 | *Clostridium sp. str. P4-6* | 87.62 | Peat | Unpublished | [EF031001.1](http://www.ncbi.nlm.nih.gov/nucleotide/117163747?report=genbank&log$=nucltop&blast_rank=1&RID=7PT9E6MP01N) | Uncultured bacterium clone A136 | 100 | *Sus scrofa -*  Weaning piglet  (colon digesta) | Unpublished |
| 10423 | Unclassified Clostridiales | HM004597.1 | *Caloramator sp. str. TC17* | 87.94 | Hot springs, Yunnan Province, China | Unpublished | [JQ307350.1](http://www.ncbi.nlm.nih.gov/nucleotide/379062778?report=genbank&log$=nucltop&blast_rank=1&RID=7PTFWVW9014) | Uncultured bacterium clone 59-4-F9 | 100 | *Homo sapiens*  (faeces) | Unpublished |
| 11957 | Unclassified Clostridiales | AJ229251.1 | *Clostridium sp. str. FCB90-3* | 88.86 | Methanogenic bioreactor |  | [GQ358482.1](http://www.ncbi.nlm.nih.gov/nucleotide/254212006?report=genbank&log$=nucltop&blast_rank=1&RID=7PTM6MF7014) | Uncultured Clostridiales bacterium clone SHTP596 | 99 | *Macropus eugenii* (forestomach sample) |  |
| 22866 | Unclassified Clostridiales | AY312403.2 | *Alkalibacter saccharofermentans str. Z-79820* | 86.29 | Soda lake, Russia | Unpublished | [GQ358429.1](http://www.ncbi.nlm.nih.gov/nucleotide/254211953?report=genbank&log$=nucltop&blast_rank=1&RID=7PTT0NPT016) | Uncultured Clostridiales bacterium clone SHTP406 | 99 | *Macropus eugenii*  (forestomach sample) |  |
| 7560 | Unclassified Lachnospiraceaea | AJ518873.1 | *Lachnobacterium sp. st. wal 14165* | 98.58 | Homo sapiens (faeces) | Unpublished | [GQ358436.1](http://www.ncbi.nlm.nih.gov/nucleotide/254211960?report=genbank&log$=nucltop&blast_rank=1&RID=7PTWH3RH016) | Uncultured Lachnospiraceae bacterium clone SHTP424 | 99 | *Macropus eugeni* (forestomach content) |  |
| 14134 | Unclassified Lachnospiraceae | ACFX02000046 | *Clostridium sp. str. M62/1* | 96.90 | *Homo sapiens* (colon) | Unpublished | [GQ297844.1](http://www.ncbi.nlm.nih.gov/nucleotide/242250842?report=genbank&log$=nucltop&blast_rank=1&RID=7PTZUP5T01N) | Uncultured bacterium clone 16saw63-2d12.w2k | 98 | *Mus musculus*  (faeces) |  |
| 23592 | Unclassified Lachnospiraceae | ACFX02000046 | *Clostridium sp. str. M62/1* | 95.71 | *Homo sapiens* (colon) | Unpublished | [GQ898730.1](http://www.ncbi.nlm.nih.gov/nucleotide/261262874?report=genbank&log$=nucltop&blast_rank=1&RID=7PU62BYV01N) | Uncultured bacterium clone S4-64 | 98 | *Homo sapiens*  (faeces) | Unpublished |
| 6013 | Unclassified Lachnospiraceae | ABOU02000049.1 | *Ruminococcus lactaris str.* ATCC 29176 | 97.38 | *Homo sapiens* (faeces) | Unpublished | [EU472616.1](http://www.ncbi.nlm.nih.gov/nucleotide/169288091?report=genbank&log$=nucltop&blast_rank=1&RID=7PUG737801N) | Uncultured bacterium clone EAC_2_aaa04f04 | 98 | *Angola colobus*  (faeces) |  |
| 20417 | Unclassified Lachnospiraceae | ACFX02000046 | *Clostridium sp. str. M62/1* | 97.67 | *Homo sapiens* (colon) | Unpublished | [AM237881.1](http://www.ncbi.nlm.nih.gov/nucleotide/125716576?report=genbank&log$=nucltop&blast_rank=1&RID=7PUKF5PE016) | Uncultured bacterium clone D1B-6.5-48h-13 | 100 | *Homo sapiens*  (faeces) |  |
| 24801 | Unclassified Lachnospiraceae | NR_026100.1 | *Clostridium celerecrescens str. DSM 5628* | 97.61 | - |  | [JN611250.1](http://www.ncbi.nlm.nih.gov/nucleotide/364538753?report=genbank&log$=nucltop&blast_rank=1&RID=7PUTK4NF014) | Uncultured bacterium clone DE05840F06CC2104D8F | 98 | *Mus musculus*  (colon) |  |
| 5460 | Unclassified Lachnospiraceae | AF202264.1 | *Syntrophococcus sucromutans str. S195* | 96.20 | *Bos sp.*  (rumen) | Unpublished | [GQ132759.1](http://www.ncbi.nlm.nih.gov/nucleotide/253762946?report=genbank&log$=nucltop&blast_rank=1&RID=7PWJVN9X014) | Uncultured bacterium clone 02c07 | 99 | *Sus scrofa*  (ASBR reactor treating swine waste) | Unpublished |
| 9509 | Unclassified Ruminococcaceae | NR_025670.1 | *Bacteroides capillosus str. ATCC 29799* | 91.96 | - | Unpublished | [EU775505.1](http://www.ncbi.nlm.nih.gov/nucleotide/192981482?report=genbank&log$=nucltop&blast_rank=1&RID=7PYAZ56001N) | Uncultured bacterium clone GOR_aag72f08 | 98 | *Gorilla gorilla gorilla* (faeces) |  |
| 11340 | Unclassified Ruminococcaceae | Z49863.1 | *Sporobacter termitidis str. SYR* | 90.07 | *Nasutitermeslujae sp*. (Termite- digestive tract) |  | [GQ871739.1](http://www.ncbi.nlm.nih.gov/nucleotide/260103539?report=genbank&log$=nucltop&blast_rank=1&RID=7PYP517F01N) | Uncultured bacterium clone MM11 | 99 | *Anser anser domesticus*  (caecum mucosa) | Unpublished |
| 13324 | Unclassified Ruminococcaceae | X71847.1 | *Clostridium cellulolyticum* | 91.73 | - |  | [HQ716659.1](http://www.ncbi.nlm.nih.gov/nucleotide/324960301?report=genbank&log$=nucltop&blast_rank=1&RID=7R65D62Y016) | Uncultured bacterium clone T2WK15B14 | 100 | *Sus scrofa*  (faeces) |  |
| 15576 | Unclassified Ruminococcaceae | EU541437.1 | *Clostridium orbiscindens str. AIP028.07* | 91.73 | *Homo sapiens* |  | [EU468234.1](http://www.ncbi.nlm.nih.gov/nucleotide/169283709?report=genbank&log$=nucltop&blast_rank=1&RID=7R6B9HDC016) | Uncultured bacterium clone horsej_a12 | 99 | *Equus ferus caballus*  (faeces) |  |
| 15941 | Unclassified Ruminococcaceae | Z49863.1 | *Sporobacter termitidis str. SYR* | 92.20 | *Nasutitermeslujae sp.* (Termite- digestive tract) |  | [AF371795.1](http://www.ncbi.nlm.nih.gov/nucleotide/18644504?report=genbank&log$=nucltop&blast_rank=1&RID=7R6GBGK201N) | Uncultured bacterium clone p-2027-s959-5 | 99 | *Sus scrofa*  (intestine) |  |
| 22208 | Unclassified Ruminococcaceae | Z49863.1 | *Sporobacter termitidis str. SYR* | 91.73 | *Nasutitermeslujae sp.* (Termite- digestive tract) |  | [EU465744.1](http://www.ncbi.nlm.nih.gov/nucleotide/169281219?report=genbank&log$=nucltop&blast_rank=1&RID=7R6PCYX501N) | Uncultured bacterium clone AS2_aao34f01 | 99 | Ovis ammon  (faeces) |  |
| 23399 | Unclassified Ruminococcaceae | NR_025670.1 | *Bacteroides capillosus str. ATCC 29799* | 96.22 | - | Unpublished | [FP082245.1](http://www.ncbi.nlm.nih.gov/nucleotide/258686193?report=genbank&log$=nucltop&blast_rank=1&RID=7R6WAXFU01N) | 16S rDNA sequence amplified from human fecal sample | 99 | *Homo sapiens*  (faeces) |  |
| 23895 | Unclassified Ruminococcaceae | ABOU02000049.1 | *Ruminococcus lactaris str. ATCC 29176* | 96.43 | *Homo sapiens* (faeces) | Unpublished | [EU775224.1](http://www.ncbi.nlm.nih.gov/nucleotide/192981201?report=genbank&log$=nucltop&blast_rank=1&RID=7PUB0JTM016) | Uncultured bacterium clone gir_aah94f12 | 99 | Giraffe camelopardalis reticulata  (faeces) |  |
