# Supplementary material for: Shedding Light on the Microbial Community of the Macropod Foregut Using 454-Amplicon Pyrosequencing
Source: PLoS One. 2013 Apr 23;8(4):e61463. doi: 10.1371/journal.pone.0061463 (PMC3634081; doi:10.1371/journal.pone.0061463)
Supplement: Table S3 — Bacterial OTUs, identified using tb-PCA, that enable the separation of 20 wild macropod forestomach samples into host macropod species, classified using the BLAST algorithm against the Greengenes and NCBI Genbank nucleotide databases. (DOC) [file pone.0061463.s003.doc]

|  |  | **Greengenes taxonomy** | | | | | **NCBI Genbank BLAST results** | | | | |
| --- | --- | --- | --- | --- | --- | --- | --- | --- | --- | --- | --- |
| **OTU ID** | **Taxonomic Classification** | **Accession number** | **Closest named relative** | **Max identity (%)** | **Host** | **Reference** | **Accession number** | **Closest match** | **Max identity (%)** | **Host** | **Reference** |
| 16563 | Unclassified Prevotellaceae | FJ545433.1 | *Prevotella genomosp. T2 str W1435* | 92.95 | *Homo sapiens* (oral cavity) |  | [GQ358254.1](http://www.ncbi.nlm.nih.gov/nucleotide/254211778?report=genbank&log$=nucltop&blast_rank=1&RID=89D9BAA7016) | Uncultured Prevotellaceae bacterium clone SGYA422 | 97 | *Macropus eugenii*  (forestomach contents) |  |
| 18040 | Unclassified Prevotellaceae | AB547702.1 | *Prevotella oulorum str. JCM 14966* | 92.5 | - |  | [GQ358416.1](http://www.ncbi.nlm.nih.gov/nucleotide/254211940?report=genbank&log$=nucltop&blast_rank=1&RID=89GEY4K801R) | Uncultured Prevotellaceae bacterium clone SGYA749 | 99 | *Macropus eugenii*  (forestomach contents) |  |
| 12266 | Unclassified Prevotellaceae | AB200227.1 | *Bacteroides helcogenes str. JCM 6927* | 84.58 | - |  | [GQ358377.1](http://www.ncbi.nlm.nih.gov/nucleotide/254211901?report=genbank&log$=nucltop&blast_rank=1&RID=89GHZNT701R) | Uncultured Bacteroidales bacterium clone SGYA667 | 97 | *Macropus eugenii*  (forestomach contents) |  |
| 4506 | Unclassified Lachnospiraceae | AF202264.1 | *Syntrophococus sucromutans str. S195* | 97.62 | *Bos sp.*  (rumen) | Unpublished | [JQ346731.1](http://www.ncbi.nlm.nih.gov/nucleotide/378405428?report=genbank&log$=nucltop&blast_rank=1&RID=89GMPTBC014) | *Syntrophococcus sucromutans* strain S195 | 98 | *Bos taurus*  (rumen) |  |
| 3363 | Unclassified Prevotellaceae | AB547678.1 | *Prevotella dentalis str. JCM 13448* | 92.73 | - |  | [GQ358346.1](http://www.ncbi.nlm.nih.gov/nucleotide/254211870?report=genbank&log$=nucltop&blast_rank=1&RID=89GUG8AY014) | Uncultured Prevotellaceae bacterium clone SGYA601 | 97 | *Macropus eugenii*  (forestomach contents) |  |
| 14760 | Unclassified Bacteroidales | DQ888330.2 | *Pontibacter sp. str. z1* | 84.21 | - |  | [EU776243.1](http://www.ncbi.nlm.nih.gov/nucleotide/192984221?report=genbank&log$=nucltop&blast_rank=1&RID=89GXBGFA01R) | Uncultured bacterium clone IR_aaa02c02 | 95 | *Rhinoceros unicornis*  (faeces) |  |
| 20764 | Unclassified Ruminococcaceae | AF030447.1 | *Ruminococcus flavefaciens str. 007* | 91.00 | *Alces sp.*  (rumen) | Unpublished | [EU475693.1](http://www.ncbi.nlm.nih.gov/nucleotide/169291168?report=genbank&log$=nucltop&blast_rank=1&RID=89H62YUN016) | Uncultured bacterium clone BARB_aaa03b07 | 94 | *Babyrousa babyrussa* (faeces) |  |
| 19773 | Unclassified Prevotellaceae | A547685.1 | *Prevotella histicola str. JCM 15367* | 95.68 | - |  | [GQ364685.1](http://www.ncbi.nlm.nih.gov/nucleotide/255045044?report=genbank&log$=nucltop&blast_rank=1&RID=89HAHZVN01R) | Uncultured bacterium clone 300BAL_G09 | 96 | *Homo sapiens*  (bronchoalveolar lavage) |  |
| 6527 | Unclassified Bacteroidales | AB238922.1 | *Parabacteroides distasonis str. JCM 5825* | 85.45 | - |  | [EU461503.1](http://www.ncbi.nlm.nih.gov/nucleotide/169276978?report=genbank&log$=nucltop&blast_rank=1&RID=89HG27BM01R) | Uncultured bacterium clone RH_aaj91b03 | 96 | *Diceros bicornis*  (faeces) |  |
| 21279 | Unclassified Prevotellaceae | AB547690.1 | *Prevotella maculosa str. JCM 15638* | 93.64 | - |  | [GQ358409.1](http://www.ncbi.nlm.nih.gov/nucleotide/254211933?report=genbank&log$=nucltop&blast_rank=1&RID=89HK7KPG01R) | Uncultured Prevotellaceae bacterium clone SGYA734 | 99 | *Macropus eugenii*  (forestomach contents) |  |
| 13869 | Unclassified Prevotellaceae | AY608696.1 | Bacteroides sp. str. WAL 10018 | 86.17 | - |  | [EU471832.1](http://www.ncbi.nlm.nih.gov/nucleotide/169287307?report=genbank&log$=nucltop&blast_rank=1&RID=89HP45M101R) | Uncultured bacterium clone AE2_aaa01b12 | 99 | *Elephas maximus*  (faeces) |  |

**References**

1. Holden M, Hauser H, Sanders M, Ngo T, Chervach I, et al. (2009) Rapid Evolution of Virulence and Drug Resistance in the Emerging Zoonotic Pathogen *Streptococcus suis*. PLoS ONE 4: e6072.

2. Boyle B, Vaillancourt K, Bonifait L, Charette SJ, Gottschalk M, et al. (2012) Genome sequence of the swine pathogen *Streptococcus suis* serotype 2 strain S735. Journal of Bacteriology 194: 6343-6344.

3. Schmidt B, Mulder IE, Musk C, Aminov RI, Lewis M, et al. (2011) Establishment of Normal Gut Microbiota Is Compromised under Excessive Hygiene Conditions. PLoS ONE 6: E28284.

4. Li E, Hamm CM, Gulati AS, Sartor RB, Chen H, et al. (2012) Inflammatory Bowel Diseases Phenotype, *C. difficile* and NOD2 Genotype Are Associated with Shifts in Human Ileum Associated Microbial Composition. PLoS ONE 7: E26284.

5. Durso LM, Harhay GP, Smith TP, Bono JL, Desantis TZ, et al. (2010) Animal-to-animal variation in fecal microbial diversity among beef cattle. Applied and Environmental Microbiology 76: 4858-4862.

6. Wallace JR, Chaudhary LC, McKain N, McEwan NR, Richardson AJ, et al. (2006) *Clostridium proteoclasticum*: A ruminal bacterium that forms stearic acid from linoleic acid. FEMS Microbiology Letters 265: 195-201.

7. Ouwerkerk D, Klieve AV, Forster RJ, Templeton JM, Maguire AJ (2005) Characterization of culturable anaerobic bacteria from the forestomach of an eastern grey kangaroo, *Macropus giganteus*. Letters in Applied Microbiology 41: 327-333.

8. Ludwig W, Weiznegger M, Dorn S, Adreesen J, Schleifer K-H (1990) The phylogenetic position of *Peptococcus niger* based on 16S rRNA sequence studies. FEMS Microbiology Letters 71: 139-144.

9. Ley RE, Hamady M, Lozupone C, Turnbaugh PJ, Ramey RR, et al. (2008) Evolution of mammals and their gut microbes. Science 320: 1647-1651.

10. Iiono T, Mori K, Tanaka K, Suzuki K, Harayama S (2007) *Oscillibacter valericigenes gen. nov., sp. nov*., a valerate-producing anaerobic bacterium isolated from the alimentary canal of a Japanese corbicula clam. International Journal of Systematic Evolutionary Microbiology 57: 1840-1845.

11. Gourgue-Jeannot C, Kalmokoff M, Kheradpir E, Kwan J, Lampi B, et al. (2006) Dietary fructooligosaccharides alter the cultivable faecal population of rats but do not stimulate the growth of intestinal bifodobacteria. Canadian Journal of Microbiology 52: 924-933.

12. Durban A, Abellan JJ, Jimenez-Hernandez N, Ponce M, Ponce J, et al. (2011) Assessing gut microbial diversity from faeces and rectal mucosa. Microbial Ecology 61: 123-133.

13. Shiratori H, Ikeno H, Ayame S, Kataoka N, Miya A, et al. (2006) Isolation and characterization of a new *Clostridium sp.* that performs effective cellulosic waste digestion in a thermophilic methanogenic bioreactor. Applied and Environmental Microbiology 72: 3702-3709.

14. Pope PB, Denman SE, Jones M, Tringe SG, Barry K, et al. (2010) Adaptation to herbivory by the Tammar wallaby includes bacterial and glycoside hydrolase profiles different from other herbivores. Proceedings of the National Academy of Sciences of the United States of America 107: 14793-14798.

15. Lawley TD, Clare S, Walker AW, Goulding D, Stabler RA, et al. (2009) Antibiotic treatment of *Clostridium difficile* carrier mice trigger a supershedder state, spore-mediated transmission, and severe disease in immunocomprimised hosts. Infectious Immunity 77: 3661-3669.

16. Leitch EC, Walker AW, Duncan SH, Holtrop G, Flint HJ (2007) Selective colonization of insoluble substrates by human faecal bacteria. Environmental Microbiology 9: 667-679.

17. Rainey FA, Stackebrandt E (1993) 16S rDNA nalysis reveals phylogenetic diversity among polysachharolytic clostridia. FEMS Microbiology Letters 113: 125-128.

18. Harrell L, Wang Y, Antonopoulos D, Young V, Lichtenstein L, et al. (2012) Standard colonic lavage alters the natural state of mucosal-associated microbiota in the human colon. PLoS ONE 7: E32545.

19. Grech-Mora I, Fardeau M, Patel BKC, Ollivier B, Rimbault A, et al. (1996) Isolation and characterization of *Sporobacter termitidis gen. nov., sp. nov.*, from the digestive tract of the wood-feeding termite *Nasutitermeslujae*. International Journal of Systematic Bacteriology 46: 512-518.

20. Kalmokoff M, Waddington LM, Thomas M, Liang KL, Ma C, et al. (2011) Continuous feeding of antimicrobial growth promoters to commercial swine during the growing/finishing phase does not modify faecal community erythromycin resistance or community structure. Journal of Applied Microbiology 110: 1414-1425.

21. Carlier JP, Bedora-Fuare M, K'ouas G, Alauzet C, Mory F (2010) Proposal to unify *Clostridium orbiscindens* Winter et al. 1991 and *Eubacterium plautii* (Senguin 1928) Hofstad and Aasjord 1982, with description of *Flavonifractor plautii gen. nov., comb. nov.*, and reassignment of *Bacteroides capillosus* to *Pseudoflavonifractor capillosus gen. nov., comb. nov.* International Journal of Systematic Evolutionary Microbiology 60: 585-590.

22. Leser TD, Amenuvor JZ, Jensen TK, Lindecrona RH, Boye M, et al. (2002) Culture-Independent Analysis of Gut Bacteria: the Pig Gastrointestinal Tract Microbiota Revisited. Applied and Environmental Microbiology 68: 673-690.

23. Tap J, Mondot S, F. L, Pelletier E, Caron C, et al. (2009) Towards the human intestinal microbiota phylogenetic core. Environmental Microbiology 11: 2574-2584.

24. Downes J, Wade W (2011) *Prevotella fusca sp. nov.* and *Prevotella scopos sp. nov.*, isolated from the human oral cavity. International Journal of Systematic Evolutionary Microbiology 61: 854-858.

25. Sakamoto M, Ohkuma M (2010) Usefulness of the hsp60 gene for the identification and classification of Gram-negative anaerobic rods. Journal of Medical Microbiology 59: 1293-1302.

26. Kitahara M, Sakamoto M, Ike M, Sakata S, Benno Y (2005) *Bacteroides plebeius sp. nov.* and *Bacteroides coprocola sp. nov.*, isolated from human faeces. International Journal of Evolutionary Microbiology 55: 2143-2147.

27. Krumholz LR, Bryant MP (1986) *Syntrophococcus sucromutans* sp. nov. gen. nov. uses carbohydrates as electron donors and formate, methoxymonobenzenoids or *Methanobrevibacter* as electron acceptor systems. Archives of Microbiology 143: 313-318.

28. Zhang L, Zhang Q, Luo X, Tang Y, Dai J, et al. (2008) *Pontibacter korlensis sp. nov.*, isolated from the desert of Xinjiang, China. International Journal of Evolutionary Microbiology 58: 1210-1214.

29. Hilty M, Burke C, Pedro H, Cardenas P, Bush A, et al. (2010) Disordered microbial communitiess in asthmatic airways. PLoS ONE 5: E8578.

30. Sakamoto M, Benno Y (2006) Reclassification of *Bacteroides distasonis*, *Bacteroides goldsteinii* and *Bacteroides merdae* as *Parabacteroides goldensteinii comb. nov.* and *Parabacteroides merdae comb. nov.* International Journal of Evolutionary Microbiology 56: 1599-1605.

31. Song YL, Liu CX, McTeague M, Finegold SM (2004) '*Bacteroides nordii*' *sp. nov.* and '*Bacteroides salyersae*' *sp. nov.* isolated from clinical specimens of human intestinal origin. Journal of Clinical Microbiology 42: 5565-5570.
